# Substance abuse treatment in Nigeria: applying a biopsychosocial-spiritual framework at MACCARCA

**DOI:** 10.3389/fpsyg.2025.1639570

**Published:** 2025-10-17

**Authors:** Mary Frances Ezeakunne, Human-Friedrich Unterrainer

**Affiliations:** ^1^Mater Christi Human Development and Religious Formation Centre, Amawbia, Nigeria; ^2^Faculty of Psychotherapy science, Sigmund Freud University Vienna, Vienna, Austria; ^3^Center for Integrative Addiction Research, Grüner Kreis Society, Vienna, Austria; ^4^Department of Religious Studies, University of Vienna, Vienna, Austria; ^5^Department of Psychiatry and Psychotherapeutic Medicine, Medical University of Graz, Graz, Austria

**Keywords:** addiction treatment, biopsychosocial-spiritual model, integrated care, Nigeria, rehabilitation, spirituality, substance abuse, traditional healing

## Abstract

**Introduction:**

Substance use disorders are a growing public-health problem in Nigeria where many existing treatments are fragmented and heavily influenced by spiritual/traditional approaches. The biopsychosocial-spiritual (BPSS) model offers an integrative framework that explicitly incorporates spiritual care alongside biological, psychological, and social interventions.

**Methods:**

We present a practice-based descriptive report supported by a single illustrative case from the Mater Christi Counselling and Rehabilitation Centre (MACCARCA), Amawbia, Nigeria. Clinical screening and assessment tools employed at MACCARCA (e.g., ASI, ASSIST, AUDIT/DAST, selected projective/personality instruments, and relevant laboratory testing) guided individualized BPSS treatment plans; outcomes are reported descriptively.

**Results:**

The case (“Romanus”) demonstrated clinically meaningful improvements across biological (stabilization, improved nutrition), psychological (reduced cravings, improved insight), social (reengagement with family, group support), and spiritual (renewed meaning and participation in spiritual practices) domains following three months of BPSS-based care. Improvements were documented in clinical notes and structured assessments and supported by patient self-report and family feedback.

**Discussion:**

The BPSS framework enabled coordinated, culturally congruent care that addressed multiple drivers of addiction and contrasted with earlier ineffective traditional/religious interventions. Limitations include the single-case design and restricted generalizability; the report is presented as a practice-based step toward larger empirical evaluation.

**Conclusion:**

The BPSS approach shows promise as a culturally adaptable, holistic model for addiction care in Nigeria. The manuscript calls for systematic outcome studies and policy actions to integrate BPSS components into broader services.

## 1 Introduction

Substance abuse, or addiction, refers to the intense and uncontrollable craving for psychoactive substances such as alcohol and illicit drugs, leading to persistent use despite harmful consequences (National Institute on Drug Abuse, 2018; Umejiaku et al., 2023). In this article, substance abuse is used synonymously with Substance Use Disorder (SUD), which is characterized by cognitive, behavioral, and physiological symptoms that persist despite negative outcomes (American Psychiatric Association, 2013). The pathological use of psychoactive substances is a global public health concern. In Nigeria, the widespread abuse of these substances poses serious threats to all sectors of life (Hussaini and Abbas, 2023), undermining the economic development, public health, religious institutions, national security, and peace (Agwogie, 2022). In 2018, the South East Zone of Nigeria comprising Abia, Imo, Anambra, Enugu, and Ebonyi States-recorded approximately 1.55 million drug users (Umejiaku et al., 2023). The 2019 Nigerian Cannabis Cultivation Survey, conducted by the National Drug Law Enforcement Agency (NDLEA), the United Nations Office on Drugs and Crime (UNODC) Country Office in Nigeria, and the UNODC Research and Trend Analysis Branch, revealed widespread domestic cultivation and international export of cannabis. Cannabis remains the most commonly used drug in the country. According to the 2018 UNODC Drug Use Survey, more than 10% of the adult population—about 10.6 million people—reported using cannabis within the previous year. Over half of all individuals admitted to treatment for illicit drug use in 2018 were treated for cannabis-related issues (United Nations Office on Drugs Crime, 2022). Other substances are also widely abused in Nigeria. The 2018 UNODC survey reported that 4.6 million Nigerians misused pharmaceutical opioids such as tramadol, while 2.4 million reported amphetamine use (United Nations Office on Drugs Crime, 2022). Alcohol misuse also remains a significant but underreported concern.

Due to the harmful effects of psychoactive substance use in Nigeria, various intervention efforts have emerged to mitigate or eliminate the problem. As substance abuse has become a major national concern, government administrations have sought stringent and sustainable measures to address the growing burden (Orukwowu, 2022; Umejiaku et al., 2023). In response, several agencies and policy frameworks have been developed as part of the government's long-standing fight against illicit drug use. However, despite these efforts, the prevalence of substance abuse and the circulation of illegal drugs continue to rise. In addition to the federal drug laws and national strategies, various institutions have been established to combat substance abuse. These include psychiatric hospitals, rehabilitation centers, prayer ministry centers, and traditional healing centers. These institutions play a vital role in supporting individuals struggling with addiction and reflect a range of treatment options available across cultural, medical, and spiritual domains (Orukwowu, 2022). Beyond Nigeria, other African countries adopt pluralistic approaches to addiction care. In Ghana, for instance, psychiatric hospitals, faith-based prayer camps, and herbal treatment centers operate side by side, often reflecting indigenous cosmologies and Christian practices (Ae-Ngibise et al., 2010). Similarly, in South Africa, community-based organizations integrate Western psychotherapy with traditional healing (Audet et al., 2017). These overlaps illustrate that while local expressions differ, the challenge of balancing cultural relevance with evidence-based care is shared across the continent. In the Nigerian context, substance abuse has been approached predominantly through spiritual or moral frameworks, often neglecting biological and psychosocial determinants (Orukwowu, 2022). Biologically, addiction alters brain reward pathways; psychologically, it involves maladaptive coping and trauma; socially, it is reinforced by stigma, unemployment, and peer influence; and spiritually, it may involve existential crises or maladaptive religiosity (Unterrainer et al., 2013). Earlier models in Nigeria often emphasized only one dimension—typically the spiritual—thereby oversimplifying a complex phenomenon. The BPSS model addresses this gap by integrating all four dimensions within a culturally adapted but evidence-based framework (Egunjobi, 2016).

This practice-based descriptive report, supported by a detailed case study, discusses the application of the Biopsychosocial-Spiritual (BPSS) model in treating substance abuse within a rehabilitation setting in Nigeria. The BPSS approach is holistic, addressing the biological, psychological, social, and spiritual aspects of individuals affected by substance abuse. A case example will be given in order to further illustrate this. The paper also reviews studies by Ebiti and Ike (2019), which examined traditional and religious treatment methods used in twenty seven Nigerian rehabilitation centers. Detailed discussions of these traditional and religious approaches will be provided. Additionally, the paper offers a critical evaluation of these treatment methods within the BPSS framework, highlighting their strengths and limitations. Finally, future prospects for integrating traditional and religious treatments with Western therapeutic approaches are explored, aiming to enhance the effectiveness and cultural relevance of substance abuse interventions in Nigeria.

### Traditional and religious treatment of substance abuse in Nigeria

Ebiti and Ike (2019) investigated traditional and religious substance abuse treatments in Northwestern and Southwestern Nigeria, analyzing twenty seven inpatient centers. These centers used religious (Christian/Islamic), traditional, or combined methods, with 82% preferring hybrid approaches. Most centers attributed addiction to spiritual causes: 26 to spiritual forces, 25 to demonic possession, 18 to moral failure, 8 to curses, and only 3 to psychological issues. One cited spiritual warfare (Ebiti and Ike, 2019). Many centers used prayer; divination and sacred rituals. Corporal punishment, restraints, and forced labor were widespread. Herbal concoctions, often with Islamic texts were used in 18 centers, sometimes mixed with drugs for detoxification. Qur'an memorization occurred in 17 centers; other practices included ritual baths and shaving (Ebiti and Ike, 2019). However, no center maintained treatment records. This lack of documentation limits knowledge transfer and evaluation (Madu, 2024; Nwoko, 2020; World Health Organization, 2002). Practitioners lacked medical training, causing dosage and safety concerns (Nwankwo, 2023; Ahime, 2022; World Health Organization, 2002). Infrastructure was poor, and ethical issues like flogging and chaining were common (Madu, 2024). Seventeen centers taught only Islamic education, risking religious bias. Overreliance on spiritual causation oversimplifies addiction, often ignoring psychological and social roots (Ebiti and Ike, 2019; Madu, 2024). In many African contexts, prayer camps are a prominent form of traditional-religious intervention. These camps, often run by Pentecostal or charismatic churches, typically frame addiction as a manifestation of demonic possession or ancestral curses. Practices may include prolonged fasting, exorcisms, or ritual confinement. While culturally resonant, such methods raise significant concerns regarding human rights, documentation, and medical oversight (Sorsdahl et al., 2010).

The BPSS model offers a holistic, evidence-based alternative, addressing biological, psychological, social, and spiritual factors (Egunjobi, 2016; Plessis, 2018; Unterrainer et al., 2013). It shares some elements with traditional methods but emphasizes empirical rigor and ethical standards. Traditional methods are affordable and culturally resonant but face major issues: inconsistent practices, unsafe environments, and lack of referrals, leading to preventable harm (Akwash, 2017; Nwoko, 2020; Iweadighi, 2011; Ejiofor, 2016). Vulnerable patients may be exploited with harmful rituals, worsening mental health (Ejiofor, 2016). In sum, while traditional treatments remain culturally significant, their effectiveness is limited without reforms. Improved documentation, training, ethical oversight, and integration with scientific models are essential for future relevance (Ebiti and Ike, 2019).

## 2 Methods

### 2.1 Biopsychosocial-Spiritual (BPSS) treatment of substance abuse in a Nigerian rehabilitation center (MACCARCA)

Mater Christi Counseling and Rehabilitation Center, Amawbia (MACCARCA), is a registered NGO located in in Amawbia, Anambra State, in southeastern Nigeria, a region characterized by dense urban-rural mix and significant challenges related to youth unemployment and drug availability. The center thus operates within a context where both cultural resources and socioeconomic stressors shape addiction trajectories. MACCARCA was founded on December 13, 2014, it is a Catholic institution run by the Sisters of the Immaculate Heart of Mary, Mother of Christ. MACCARCA provides holistic care—addressing the biological, psychological, social, and spiritual needs of its clients. Its mission is to offer healing and empowerment, helping individuals take responsibility for their lives. The center offers both inpatient and outpatient services, admitting clients as needed or supporting them through regular visits. Clients with the following clinical problems receive treatment at this center: anxiety disorder, panic attack, depression, personality disorders, schizophrenia, substance abuse, children with behavioral misconduct, children with academic challenges, clients with hyperactive and attention deficit problems, etc. More so, families and couples with relational problems and marital challenges receive psychotherapy at this center. In addition to the treatment of clients with some pathologies or disorders, MACCARCA is known for the administration of psychological evaluations to different people (see Supplementary file for some illustrative images of MACCARCA). MACCARCA was selected as the focal site because it is one of the few centers in southeastern Nigeria that systematically applies an integrative BPSS framework. Although one of the authors is affiliated with the center, this affiliation enabled accurate documentation of treatment processes. We clarify that the aim is not to advertise the center but to provide a practice-based account that may inform broader treatment discourse. A conflict-of-interest statement has been added to make this transparent.

### 2.2 Therapeutic approaches at MACCARCA

**Figure 1:**
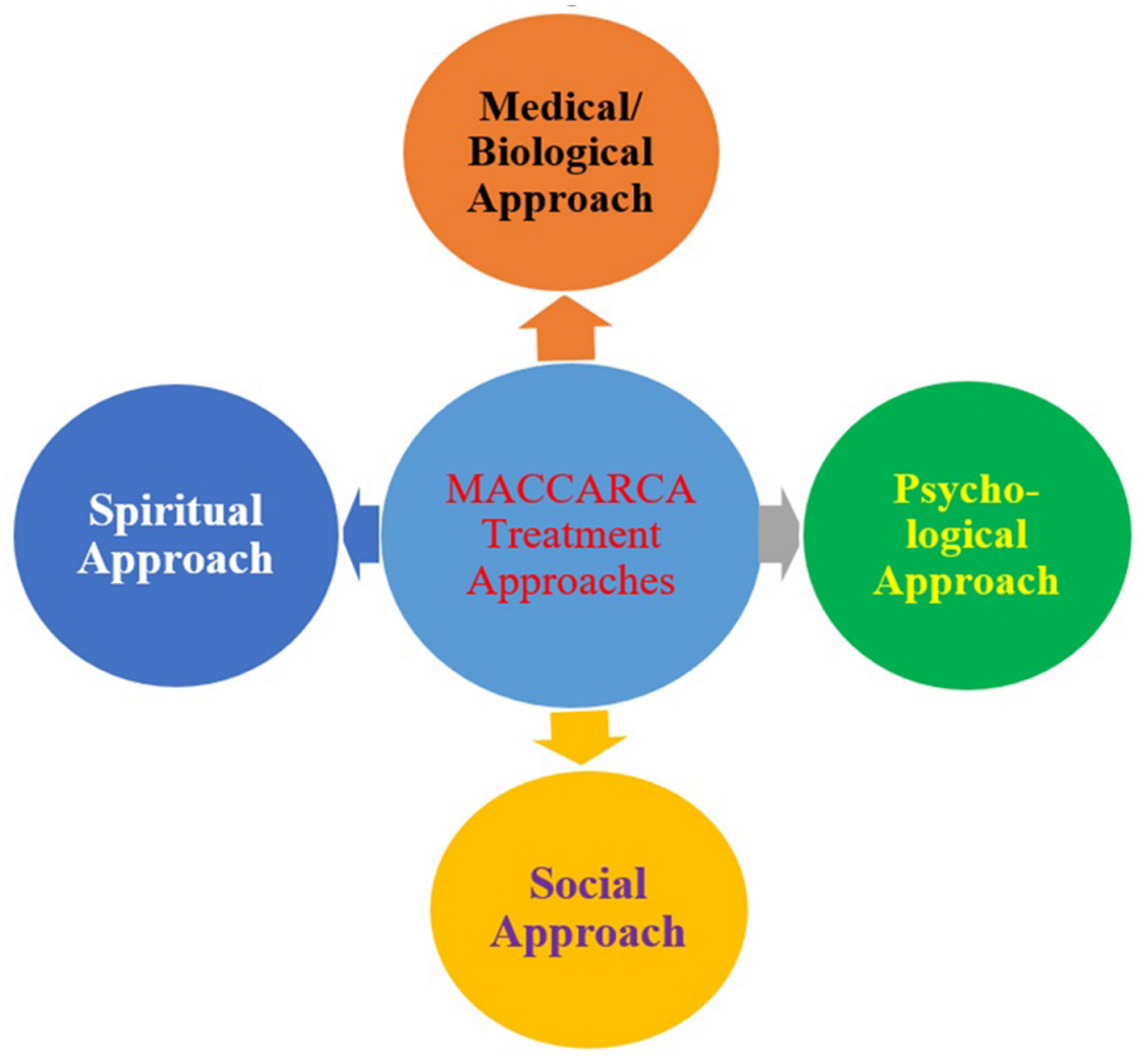
Medical/biological, psychological, social, and spiritual treatment.

MACCARCA offers a range of treatment methods, including individual, group, couple, and family therapy. Individual and group sessions are held daily, while couple and family therapies occur less frequently. The center uses various psychotherapeutic approaches such as psychodynamic therapy, Cognitive Behavioral Therapy (CBT), client-centered therapy, rational emotive behavioral therapy (REBT), relational, and supportive therapies. The core treatment model at MACCARCA is the BPSS approach, which views each client holistically—addressing physical, psychological, social, and spiritual needs. This model is especially applied to clients struggling with substance abuse and involves a multidisciplinary team providing integrated care. All clients undergo a BPSS assessment upon admission to identify key areas contributing to their challenges. This assessment forms the basis for a tailored treatment plan. The four pillars of treatment—medical/biological, psychological, social, and spiritual—are designed to work together to support lasting healing and recovery (see Figure 1 for further illustration).

#### 2.1.1 Medical/biological treatment approach

The medical/biological approach is a key component of treatment at MACCARCA. It begins with a biological assessment, including lab tests such as liver and kidney function, HIV, malaria, hepatitis B and C, full blood count, and ECG, conducted at a nearby diagnostic center. These assessments guide decisions on medical care, though not all clients require medication. In cases involving severe psychosis (e.g., delusions, hallucinations) or substance withdrawal, medication is often essential to stabilize the client before therapy can be effective. Psychiatrists prescribe appropriate medications based on individual diagnoses, and in cases of withdrawal, treatment includes medically supervised detoxification, regular nursing care, and general medical review. Nutritional therapy is also an important part of biological care at MACCARCA. Many clients, particularly those with substance use disorders, suffer from poor nutrition due to erratic eating habits. Substances like alcohol and stimulants can suppress appetite and impair nutrient absorption. To aid recovery, clients receive balanced meals rich in fruits, vegetables, vitamins, and minerals, helping to restore brain chemistry and overall health. This combined medical and nutritional support enhances clients' ability to engage in therapy and recover more effectively.

In addition to medication and nutrition, physical activities and outdoor games are key aspects of biological treatment at MACCARCA. The center offers various sports such as football, handball, brisk walking, dancing, yoga, table tennis, and rope skipping. These activities, usually scheduled in the morning, support physical and mental wellbeing. MACCARCA also provides a fitness facility equipped with gym equipment, allowing clients to follow regular exercise routines that promote both physical and emotional health. Gardening, or horticultural therapy, is another important activity. Clients engage in planting and weeding vegetables and flowers during community service. This connection with nature has therapeutic benefits and promotes relaxation. The informal setting of gardening, often shared with therapists or staff, encourages open interaction, helping clients feel more at ease and supported in their healing process.

#### 2.1.2 Psychological treatment approach

At MACCARCA, psychological care begins with a comprehensive evaluation by clinical psychologists as part of the BPSS model. This includes in-depth interviews and psychological assessments that explore the client's personality style or disorder, cognitive and emotional functioning, behavioral patterns, and relational issues. The resulting psychological report provides valuable insight into the client's self-image, emotional states (such as anger, fear, guilt, or low self-esteem), and defense mechanisms—whether mature, neurotic, or primitive. It also reveals underlying conflicts and traumas, both intra-psychic and inter-psychic, that may be influencing current behaviors. With this information, therapists can tailor treatment effectively. The goal of psychotherapy is not only to alleviate symptoms but to promote long-term personality growth and healthier coping strategies (Oliver, 2021).

Working with clients who struggle with addiction can be particularly challenging. Many lack insights or deny the negative consequences of substance use. They often rely on ego defense mechanisms such as denial, projection, rationalization, or intellectualization. These distorted thought patterns prevent them from recognizing their problems. As May (1988) notes, clients may believe they can control their lives through substance use. Therapy aims to dismantle this illusion through psychological assessment and individualized psychotherapy, creating opportunities for meaningful change. Individual therapy provides a safe space for clients to reflect and discuss personal issues, including those raised in group therapy. In some cases, family involvement becomes necessary. MACCARCA offers family therapy sessions, acknowledging that a client's healing is influenced by their home environment. Involving family in therapy helps therapists understand their contributing factors to the client's condition and enhances long-term recovery. Constructing a genogram during family sessions is a key tool for tracing generational patterns of psychological, physical, and spiritual health or dysfunction (Sperry, 2001). Periodically, MACCARCA holds open day sessions where family members can visit, participate in discussions, and track the client's progress as part of a supportive recovery process.

#### 2.1.3 Social treatment approach

At MACCARCA, social assessment is an integral part of the BPSS evaluation. It aims to understand the client's social support system, cultural beliefs, and relationships with family and peers. This helps determine the appropriate social interventions each client may need. Social interventions are carried out by social therapists, occupational therapists, psychologists, and occasionally other trained staff. These interventions often involve group processes and community support. Individuals struggling with substance use disorders frequently face stigma and negative perceptions from both their families and society, leading to feelings of shame, isolation, and alienation (Yang et al., 2018).

Social support and group therapy play a crucial role in helping clients regain a sense of inclusion, empowerment, and safety. According to Zaidi (2020), addiction clients involved in supportive group therapy are better equipped to manage their psychological and substance use issues. Group therapy helps reintegrate clients into a community that accepts them unconditionally, serving as a powerful agent of change. Through group dynamics, clients receive emotional support and constructive feedback, reducing isolation and fostering trust. At MACCARCA, both formal sessions and informal social gatherings are part of the therapeutic environment. Clients are encouraged to engage with others, except when therapeutic solitude is needed for rest and recovery.

MACCARCA employs a comprehensive, socially oriented therapeutic approach that includes group therapy, where clients share experiences and find mutual support in homogeneous, heterogeneous, or gender-based groups. Psycho-educational therapy teaches emotional regulation, life skills, and addiction recovery, while bibliotherapy uses relevant literature to inspire reflection for those who can read. Recreational therapy offers structured, relaxing social activities, and film therapy utilizes motivational or therapeutic films to promote discussion and insight. Clients also engage in community service, fostering responsibility and reconditioning passive behavior, with some later employed or supported in education or work. Vocational training equips clients with practical skills—such as catering, sewing, computer literacy, or cosmetology—helping them stay engaged and build economic independence. Together, these therapies support clients in healing and rebuilding their lives with purpose and dignity.

#### 2.1.4 Spiritual treatment approach

Spiritual treatment is one of the key interventions at MACCARCA, emphasizing spiritual principles and offering guidance to substance use disordered clients seeking healing through spirituality. This approach focuses on individuals' relationships with God and their spiritual wellbeing (see e.g., Cook, 2004 or Galanter, 2006 for further discussion). At MACCARCA, spiritual care is overseen by pastoral therapists or spiritual guides, with a chaplain conducting daily Mass and regular confessions. Clients with special spiritual needs may receive individualized support, and those of other denominations are allowed to see their own spiritual leaders upon request. Spirituality has been widely recognized as an important therapeutic component (Galanter et al., 2023). Youssef (2022) notes that incorporating spirituality into counseling helps individuals regain mental health and find meaning and purpose. Therefore, pastoral therapists at MACCARCA are encouraged to adopt a non-denominational, inclusive approach that respects diverse beliefs. As Cook (2009) categorizes, substance misuse spiritual programs generally fall into three types: (1) 12-step spirituality, (2) spirituality rooted in faith traditions, and (3) non-specific spiritual practices. All three are utilized at MACCARCA (see Youssef, 2022). MACCARCA serves clients from diverse religious backgrounds. Those from Protestant, Pentecostal, or Muslim traditions are encouraged to continue their own practices and, when possible, are supported by visits from their spiritual leaders. This inclusivity minimizes conflict between denominational practices and ensures spiritual care is client-centered rather than prescriptive.

Many clients choose MACCARCA for therapy because of its spiritual dimension, which acknowledges the addict's powerlessness and emphasizes God's healing grace. The 12-step program, a non-denominational and inclusive model, is central to MACCARCA's spiritual therapy (Dermatis and Galanter, 2016). Participation in spiritual practices is entirely voluntary and never imposed. As part of the BPSS model, spiritual assessment at MACCARCA explores a client's relationship with God, the impact of their religious affiliation on their worldview, and their interactions with others. For instance, some clients equate loving God with extreme fasting, which may contribute to health issues. Spiritual assessment also helps identify unhealthy spiritual expressions such as religious delusions, scrupulosity, excessive guilt, or shame. Beyond questions about God, spiritual evaluation delves into what gives a person meaning and purpose (Culliford and Eagger, 2009). It examines the client's essence—beliefs, values, motivations, and attachments. This understanding is crucial to shaping effective spiritual therapy. Spiritual interventions play a key role in relapse prevention, helping clients connect with their identity and life's purpose. They also promote the development of strong moral and religious values, supporting clients in leading healthier and more meaningful lives.

### 2.3 Practical application of BPSS interventions on clients with substance abuse at MACCARCA

The general treatment strategies at MACCARCA have been outlined above. This section focuses specifically on clients with substance abuse issues. While they also receive BPSS-based care, certain processes are unique to their treatment. MACCARCA emphasizes recovery and prevention—not substitution or harm reduction. Treatment is divided into four key phases: screening and biopsychosocial-spiritual assessment, detoxification, rehabilitation, and discharge with post-treatment (aftercare) support.

### Phase 1: Assessment

Upon arrival at MACCARCA, clients with substance abuse issues undergo a comprehensive assessment tailored to their symptoms and substance use history. This first phase includes:

#### 2.3.1 Biopsychosocial-spiritual assessment

This comprehensive evaluation covers the client's biological, psychological, social, and spiritual wellbeing. It includes an in-depth interview and may involve standardized psychological tests such as the MMPI, TAT, Rorschach, Rotter Incomplete Sentences Blank, Millon Clinical Multiaxial Inventory, and the Self-Esteem Inventory.

#### 2.3.2 Screening

Clients are screened to determine the severity and impact of substance use using tools such as the Addiction Severity Index (ASI), Alcohol, Smoking and Substance Involvement Screening Test (ASSIST), Drug Abuse Screening Test (DAST), Alcohol Use Disorders Identification Test (AUDIT and AUDIT-C), Methamphetamine Use Scale (MUS), and Trauma Symptom Inventory-2 (TSI-2)

These tools were used clinically to assess the severity of substance use, psychiatric comorbidities, and psychosocial functioning. They informed individualized treatment planning rather than generating quantitative data for statistical analysis. As this is a descriptive case report, results from these measures are presented narratively to illustrate the application of the BPSS model in practice.

#### 2.3.3 Laboratory tests

Depending on the client's medical and substance use history, laboratory tests may include toxicology screening to identify substances used. Additional tests may involve a full blood count, liver and kidney function tests, HIV screening, hepatitis B and C panels, malaria parasite tests, blood sugar levels, urinalysis, and electrocardiogram (ECG).

### Phase 2: Detoxification

From the initial assessments, clients with physical dependence and severe withdrawal symptoms are identified and first undergo detoxification. This phase precedes the full BPSS assessment when necessary. Withdrawal symptoms vary by individual—some experience intense reactions, while others have milder symptoms—so treatment is administered symptomatically. During detoxification, psychiatrists provide medications (oral or injectable) to stabilize clients before rehabilitation begins. Clients are temporarily housed in a separate area to ensure close monitoring. Once stabilized, they are transferred to the rehabilitation unit. Detoxification helps clients achieve the calmness and clarity needed to fully engage in the center's therapeutic programs. Its duration depends on how quickly a client reaches sobriety without substance use.

### Phase 3: Rehabilitation

Only clients free from physical dependence and withdrawal symptoms proceed to the rehabilitation phase. At this point, they are no longer physically reliant on substances. After screening and detoxification, comprehensive BPSS interventions begin, incorporating medical, psychological, social, and spiritual therapies. Although some therapies may start earlier, there is no rigid sequence, as the interventions are interconnected and delivered by various professionals according to the timetable. At MACCARCA, informal therapy begins each morning when clients wake up. The day often starts with spiritual practices such as attending Mass or personal prayer, followed by breakfast, sports, and scheduled therapy sessions. Psychological care involves daily individual therapy with a designated therapist. Medically, some clients continue prescribed medications during rehabilitation. Social therapy also plays a vital role through group interaction and community participation. Spiritual therapy continues alongside other interventions. Therapists trained in spiritual care guide clients through personalized spiritual exercises to deepen healing. This integrative approach ensures that clients receive holistic support throughout their recovery journey.

### Phase 4: Discharge and post-treatment (Aftercare)

This phase is for clients who have completed the rehabilitation process. The minimum treatment duration at MACCARCA is 1 month, though some clients may stay over 6 months depending on their condition and progress. Factors like prognosis, family support, and financial capacity influence the length of stay. Unfortunately, some clients are discharged prematurely due to financial constraints, as the government does not subsidize rehabilitation costs. While MACCARCA supports some indigent clients, resources are limited. Discharge is based on the client's progress, including freedom from withdrawal symptoms or psychotic experiences, increased self-awareness of substance abuse risks, and readiness to embrace a healthier lifestyle. A client must also show confidence and plans for the future. Post-treatment (aftercare) focuses on monitoring progress, identifying signs of relapse, reviewing medications, and offering ongoing counseling. This continued support helps maintain recovery and prevent relapse. Clients typically return for scheduled appointments, although urgent or unscheduled visits are accommodated when necessary.

Building on the theoretical background and critical review of traditional and religious treatment methods in Nigeria, the following section demonstrates how the BPSS model is applied in practice at MACCARCA.

## 3 Findings

### 3.1 Case example

This case illustrates the application of the BPSS treatment model at MACCARCA. The name of the client was changed and he gave written informed consent to publish his story. Romanus was selected as an illustrative case because his treatment journey captured the full application of all four BPSS pillars. While his outcome was positive, this does not imply uniform success; rather, his case was chosen to demonstrate the model's mechanisms in practice.

### 3.2 Background

Romanus, a 23-year-old male, was the eldest of four children from a middle-income family. He completed secondary school and obtained a West African Senior Secondary Certificate (WASSC). Toward the end of his schooling, he began abusing substances—starting with tramadol and later progressing to cocaine and amphetamines, influenced by peer pressure. Unable to continue his education due to financial constraints, Romanus moved in with his maternal uncle to assist in his business. Though he tried to conceal his drug use, his uncle soon noticed behavioral changes: talkativeness, euphoria, hypersexuality, disorganized speech, and hallucinations. Romanus became increasingly aggressive and erratic, eventually abandoning the family business and engaging in risky, late-night behaviors. Initial Interventions: Upon discovering drugs in Romanus's room, his uncle informed his parents. Believing he was spiritually afflicted, they first sought help from a pastor, who conducted exorcisms over 6 months with no success. They then consulted a traditional healer who attributed the addiction to ancestral curses. Romanus was given herbal remedies and subjected to rituals, but his condition worsened. 4 years into his addiction, Romanus was arrested after a violent altercation at a bar. The police brought him to MACCARCA for rehabilitation, while the others involved fled.

### 3.3 Admission at MACCARCA

Upon arrival, Romanus was angry, disoriented, and resistant to treatment. He denied drug use and could not coherently recount his history. His parents and uncle described his recent behavior: insomnia, skipped meals, aggression, and poor hygiene—drastically different from his former self. Romanus also attempted to flee MACCARCA multiple times in search of drugs.

### 3.4 Assessment and diagnosis

Romanus underwent a detoxification process to manage withdrawal symptoms. He was then assessed using the Addiction Severity Index (ASI) and the Alcohol, Smoking and Substance Involvement Screening Test (ASSIST). These tools confirmed he met the DSM-V criteria for substance use disorder. Symptoms included: Frequent, uncontrollable drug use, cravings and withdrawal symptoms, aggression and social isolation, occupational and interpersonal dysfunction, psychotic symptoms (hallucinations).

### 3.5 Treatment plan

Once stabilized, Romanus began comprehensive BPSS therapy:

- Biological/Medical: He received medication for both substance use and psychotic symptoms. His health improved steadily.
- Psychological: Daily one-on-one sessions with a therapist using cognitive-behavioral and relational approaches. He also participated in psychoeducation, group therapy, and expressive therapies such as music, film, and recreation.
- Social: Romanus engaged in structured group activities, including sharing sessions and 12-step programs, which helped him feel supported and less isolated.
- Spiritual: Guided by a pastoral therapist, Romanus resumed religious practices, including prayer, Holy Mass, confession, and the rosary. Spiritual therapy played a key role in restoring his self-worth and inner peace.

### 3.6 Therapeutic outcomes

After 3 months at MACCARCA, Romanus showed significant improvement. He was no longer craving substances, slept and ate well, and engaged positively with staff and peers. His physical and mental health improved, and he expressed remorse for his earlier behaviors. Through therapy, he identified risk factors and triggers for his addiction, developed coping strategies to prevent relapse, rebuilt trust with his family, regained confidence and hope for the future.

### 3.7 Client reflections

Romanus credited several factors for his recovery: The welcoming, patient, and understanding attitude of MACCARCA staff, the group therapy, which helped him overcome shame and realize he wasn't alone, some activities like sports and music, which boosted his self-esteem and physical health, one-on-one therapy sessions that taught him how to manage cravings, renewed spiritual engagement, which gave him strength and perspective. The 12-step program, which offered inspiration and connection with others facing similar challenges.

### 3.8 Aftercare

Romanus was discharged after 3 months and continued aftercare through monthly visits. His family, that was once convinced his addiction was spiritual in origin, came to understand it as a medical and psychological issue requiring professional treatment.

### 3.9 Summary

Romanus's story demonstrates the effective application of the Biopsychosocial-Spiritual model in treating substance use disorder. Comprehensive assessment, individualized therapy, and integrated medical, psychological, social, and spiritual care led to positive outcomes. His case illustrates the importance of evidence-based approaches in addressing the complex nature of addiction-contrasting sharply with earlier, ineffective traditional and religious interventions.

## 4 Discussion

The BPSS model offers a holistic, evidence-based alternative, addressing biological, psychological, social, and spiritual factors. This integrative perspective reflects Engel (1977) original biopsychosocial paradigm, later expanded to include spirituality as a vital domain of health (Puchalski et al., 2009). Internationally, similar approaches have been shown to enhance outcomes in addiction treatment by fostering resilience, meaning-making, and social reintegration (Galanter et al., 2023; Miller, 2013). However, these methods face serious limitations. As World Health Organization (2002) has highlighted, the lack of standardized documentation and safety protocols undermines both quality of care and accountability. Similarly, Nwoko (2020) documents how the absence of medical training among traditional practitioners raises risks of harm, while Galanter et al. (2023) emphasize that overreliance on spiritual causation oversimplifies the complex biopsychosocial nature of addiction. This aligns with broader critiques of the biopsychosocial model, which caution against both underuse and overextension without empirical rigor (Roberts, 2023). Other integrative perspectives (Amodia et al., 2005) emphasize the necessity of spiritual and cultural dimensions in addiction care, reinforcing the rationale for BPSS in Nigeria. Studies on recovery groups (Rettie et al., 2021) and self-help organizations (Nalbantoglu and Tuncay, 2024) further demonstrate how community and spirituality serve as central components of recovery. Similarly, research on social recovery (Vigdal et al., 2022) underlines the importance of reintegration and empowerment, both of which are embedded in MACCARCA's model. Policy and practice implications emerge from this case. First, national health authorities should recognize BPSS as a viable framework and integrate it into mental health and addiction policies. Second, capacity-building for therapists and clergy should ensure ethical, evidence-based practice. Third, partnerships with community and religious institutions should be fostered to scale the model. Fourth, government subsidies and insurance coverage are essential to ensure equitable access. Finally, ongoing monitoring and evaluation systems should be developed to assess outcomes and refine practice. A key limitation of this study is its reliance on a single illustrative case, which prevents generalization of findings to broader populations. Furthermore, the study reflects the resource constraints of one rehabilitation center, where financial sustainability and limited government support affect continuity of care. These constraints highlight the need for caution when extrapolating from this case to other Nigerian or African contexts. The BPSS model's application may differ across addictions: while effective in substance-related disorders, adaptations may be needed for behavioral addictions such as gambling. Moreover, within pluralistic African settings, its implementation must navigate religious diversity, including Catholic, Pentecostal, Muslim, and African Traditional Religion perspectives. Challenges include reconciling divergent worldviews, addressing stigma, and ensuring ethical safeguards. These obstacles, however, also present opportunities for dialogue and integration, making BPSS a flexible yet culturally grounded approach.

## 5 Conclusion

This paper has highlighted the promise of the BPSS framework as an evidence-based and culturally adaptable model for the treatment of substance use disorders in Nigeria. By integrating biological, psychological, social, and spiritual dimensions, the approach addresses addiction in its full complexity and offers a viable alternative to fragmented or solely traditional interventions (Snodgrass et al., 2024; Unterrainer, 2025). While traditional and religious practices remain culturally significant, they require refinement through systematic documentation, ethical oversight, and integration with scientifically validated methods. The BPSS model demonstrates how such integration can be achieved in practice, providing both clinical effectiveness and cultural resonance (Nwankwo, 2023).

Future research should include controlled clinical trials of BPSS-based interventions across diverse populations to evaluate outcomes such as relapse rates, mental health improvements, social reintegration, and spiritual wellbeing (see Unterrainer et al., 2012, 2013 for an enhanced discussion). Such work will help establish a robust evidence base and guide policy, ensuring that addiction care in resource-limited but spiritually sensitive contexts is both effective and culturally grounded.

## Data Availability

The original contributions presented in the study are included in the article/Supplementary material, further inquiries can be directed to the corresponding author/s.

## References

[B1] Ae-Ngibise K. Cooper S. Adiibokah E. Akpalu B. Lund C. Doku V. (2010). Whether you like it or not people with mental problems are going to go to them': a qualitative exploration into the widespread use of traditional and faith healers in the provision of mental health care in Ghana. Int. Rev. Psychiatr. 22, 558–567. 10.3109/09540261.2010.536149 21226644

[B2] Agwogie M. O. (2022). Addressing drug challenges in health and humanitarian crises: settings in need of care for a comprehensive drug use prevention in Nigeria. J. Int. Soc. Subst. Use Profess. Nigeria Chapter.

[B3] Ahime U. J. (2022). Nigerian pentecostal pastors' perception of mental disorder (Unpublished doctoral dissertation). Regent University, United Kingdom.

[B4] Akwash F. B. A. (2017). Psychotherapy and traditional healing. Int. J. Psychother. Afr. 2, 117–130.

[B5] American Psychiatric Association (2013). Diagnostic and Statistical Manual of Mental Disorders, 5th Edn. Washington, DC. 10.1176/appi.books.9780890425596

[B6] Amodia D. S. Cano C. Eliason M. J. (2005). An integral approach to substance abuse. J. Psychoactive Drugs 37, 363–371. 10.1080/02791072.2005.10399809 16480163

[B7] Audet C. M. Ngobeni S. Graves E. Wagner R. G. (2017). Mixed methods inquiry into traditional healers' treatment of mental, neurological and substance abuse disorders in rural South Africa. PLoS ONE 12:e0188433. 10.1371/journal.pone.0188433 29261705 PMC5736181

[B8] Cook C. C. (2004). Addiction and spirituality. Addiction 99, 539–551. 10.1111/j.1360-0443.2004.00715.x 15078228

[B9] Cook C. C. H. (2009). “Substance misuse,” in Spirituality and Psychiatry, eds. C. C. H. Cook, A. Powell, and A. Sims (London: RCPsych Publications), 139–168.

[B10] Culliford L. Eagger S. (2009). “Assessing spiritual needs,” in Christianity and Psychiatry, eds. C. C. H. Cook, A. Powell, and A. Sims (London: Royal College of Psychiatrists Publications), 16–38.

[B11] Dermatis H. Galanter M. (2016). The role of twelve-step-related spirituality in addiction recovery. J. Religion Health 55, 510–521. 10.1007/s10943-015-0019-4 25701085

[B12] Ebiti N. W. Ike J. O. (2019). “Alternative treatment and therapies for drug addiction in Nigeria,” in Bulletin on Narcotics: Drugs in the Nigerian Population, Vol. LXII, ed. A. Me [Vienna: United Nations Office on Drugs and Crime (UNODC)], 123–134. 10.18356/da0a6cd8-en

[B13] Egunjobi J. P. (2016). Biopsychosocial-Spiritual Approach: Towards a Holistic Understanding and Treatment of Drug Addiction. Lulu Press. Available online at: https://www.amazon.co.uk/Biopsychosocial-Spiritual-Approach-Understanding-Treatment-Addiction/dp/1365121607 (Accessed September 30, 2025)

[B14] Ejiofor I. U. O. (2016). Psychotherapeutic values of Igbo healing practices: the umunna psychotherapy. Int. J. Psychother. Afr. 1, 106–121.

[B15] Engel G. L. (1977). The need for a new medical model: a challenge for biomedicine. Science 196, 129–136. 10.1126/science.847460 847460

[B16] Galanter M. (2006). Spirituality and addiction: a research and clinical perspective. Am. J. Addict. 15, 286–292. 10.1080/10550490600754325 16867923

[B17] Galanter M. White W. L. Khalsa J. Hansen H. (2023). A scoping review of spirituality in relation to substance use disorders: psychological, biological, and cultural issues. J. Addict. Dis. 42, 210–218. 10.1080/10550887.2023.2174785 36772834

[B18] Hussaini U. M. Abbas U. S. (2023). The role of religions and communities in fighting against drug abuse in Nigeria. J. Integr. Sci. 4, 174–219.

[B19] Iweadighi S. O. (2011). Sickness and the search for healing in Igboland: a pastoral theological analysis (Doctoral dissertation). University of Vienna.

[B20] Madu S. N. (2024). Practicum in Psychotherapy: An African Perspective. Enugu: Rhyce Kerex Publishers.

[B21] May G. G. (1988). Addiction and Grace: Love and *Spirituality in the Healing of Addictions*. New York, NY: HarperCollins Publishers.

[B22] Miller W. R. (2013). Addiction and spirituality. Subst. Use Misuse 48, 1258–1259. 10.3109/10826084.2013.799024 24041187

[B23] Nalbantoglu I. Tuncay T. (2024). Self-Help for Substance use disorder: a qualitative exploration of an exemplary organization in Türkiye. Alcohol. Treat. Q. 42, 481–494. 10.1080/07347324.2024.2362158

[B24] National Institute on Drug Abuse (2018). Principles of Drug Addiction Treatment: A Research-Based Guide, 3rd Edn. Bethesda, MD.

[B25] Nwankwo F. C. (2023). Sociological review of the forms, merits and challenges of the traditional health care delivery system in Nigeria. Nigerian J. Med. Sociol. 3, 34–46.

[B26] Nwoko K. C. (2020). Traditional psychiatric healing in Igbo land, southeastern Nigeria. Global J. Sociol. Anthropol. 9, 1–8.

[B27] Oliver L. (2021). An overview on psychotherapy and its significance. Clin. Psychiatr. 7:e115.

[B28] Orukwowu U. (2022). Drug abuse and the incidence of mental illness in Nigeria: a systematic review. J. Contemp. Sci. Eng. Technol. 1. Available online at: https://rajournals.net/index.php/jcset/article/view/241

[B29] Plessis G. D. (2018). An Integral Foundation for Addiction Treatment: Beyond the Biopsychosocial Model. Integral Publishers. 10.4172/1522-4821.1000406

[B30] Puchalski C. M. Vitillo R. Hull S. K. Reller N. (2009). Improving the spiritual dimension of whole person care: reaching national and international consensus. J. Palliat. Med. 12, 885–904. 10.1089/jpm.2009.0142 24842136 PMC4038982

[B31] Rettie H. C. Hogan L. M. Cox W. Miles. (2021). Identifying the main components of substance-related addiction recovery groups. Subst. Use Misuse 56, 840–847. 10.1080/10826084.2021.1899228 33745420

[B32] Roberts A. (2023). The biopsychosocial model: its use and abuse. Med. Health Care Philos. 26, 367–384. 10.1007/s11019-023-10150-2 37067677 PMC10107555

[B33] Snodgrass S. Corcoran L. Jerry P. (2024). Spirituality in addiction recovery. a narrative review. J. Religion Health 63, 515–530. 10.1007/s10943-023-01854-z 37486580

[B34] Sorsdahl K. Stein D. J. Flisher A. J. Grimsrud A. Seedat S. (2010). Traditional healers in the treatment of common mental disorders in South Africa. J. Nerv. Ment. Dis. 197, 434–441. 10.1097/NMD.0b013e3181a61dbc 19525744 PMC3233225

[B35] Sperry L. (2001). Spirituality in Clinical Practice: Incorporating the Spiritual Dimension in Psychotherapy and Counselling.New York, NY: Brunner-Routledge.

[B36] Umejiaku N. Enemchukwu R. A. Okeke O. E. (2023). The impact of drug abuse on Igbo culture and youths in Nigeria: a legal appraisal. Nnamdi Azikiwe University J. Int. Law Jurisprud. 14, 115–127.

[B37] United Nations Office on Drugs and Crime (2022). Nigeria Cannabis Survey. Available online at: https://www.unodc.org/documents/crop-monitoring/Nigeria/Nigeria_Cannabis_Survey_2022.pdf (Accessed September 30, 2025)

[B38] Unterrainer H. F. (2025). On the wings of Icarus–the need for transcendence in addictive diseases. Front. Psychiatr. 16:1563871. 10.3389/fpsyt.2025.1563871 40191112 PMC11968730

[B39] Unterrainer H. F. Huber H. P. Stelzer K. Fink A. (2012). Spiritus contra Spiritum?: spiritual wellbeing and depression among male alcohol dependents in treatment. Alcohol. Treat. Q. 30, 67–77. 10.1080/07347324.2012.635551

[B40] Unterrainer H. F. Lewis A. Collicutt J. Fink A. (2013). Religious/spiritual wellbeing, coping styles, and personality dimensions in people with substance use disorders. Int. J. Psychol. Religion 23, 204–213. 10.1080/10508619.2012.714999

[B41] Vigdal M. I. Moltu C. Bjornestad J. Selseng L. B. (2022). Social recovery in substance use disorder: a metasynthesis of qualitative studies. Drug Alcohol Rev. 41, 974–987. 10.1111/dar.13434 35104369 PMC9306622

[B42] World Health Organization (2002). WHO Traditional Medicine Strategy 2002–2005 (WHO/EDM/TRM/2002.1). Geneva: World Health Organization. Available online at: https://iris.who.int/handle/10665/67163

[B43] Yang L. Wong L. Y. Grivel M. M. Hasin D. S. (2018). Stigma and substance use disorders: an international phenomenon. Curr. Opin. Psychiatr. 30, 378–388. 10.1097/YCO.0000000000000351 28700360 PMC5854406

[B44] Youssef S. (2022). Spirituality and Psychology: Spiritual Integration in Counseling Psychology, “the Hidden Subconscious Healing Power of the Human Spirit”. Bloomington, IN: Balboa Press.

[B45] Zaidi U. (2020). Role of social support in relapse prevention for drug addicts. Int. J. Innov. Creat. Change 13, 915–924.

