# Supplementary figures and images for: Substance abuse treatment in Nigeria: applying a biopsychosocial-spiritual framework at MACCARCA
Source: Front Psychol. 2025 Oct 17;16:1639570. doi: 10.3389/fpsyg.2025.1639570 (PMC12581198; doi:10.3389/fpsyg.2025.1639570)

| **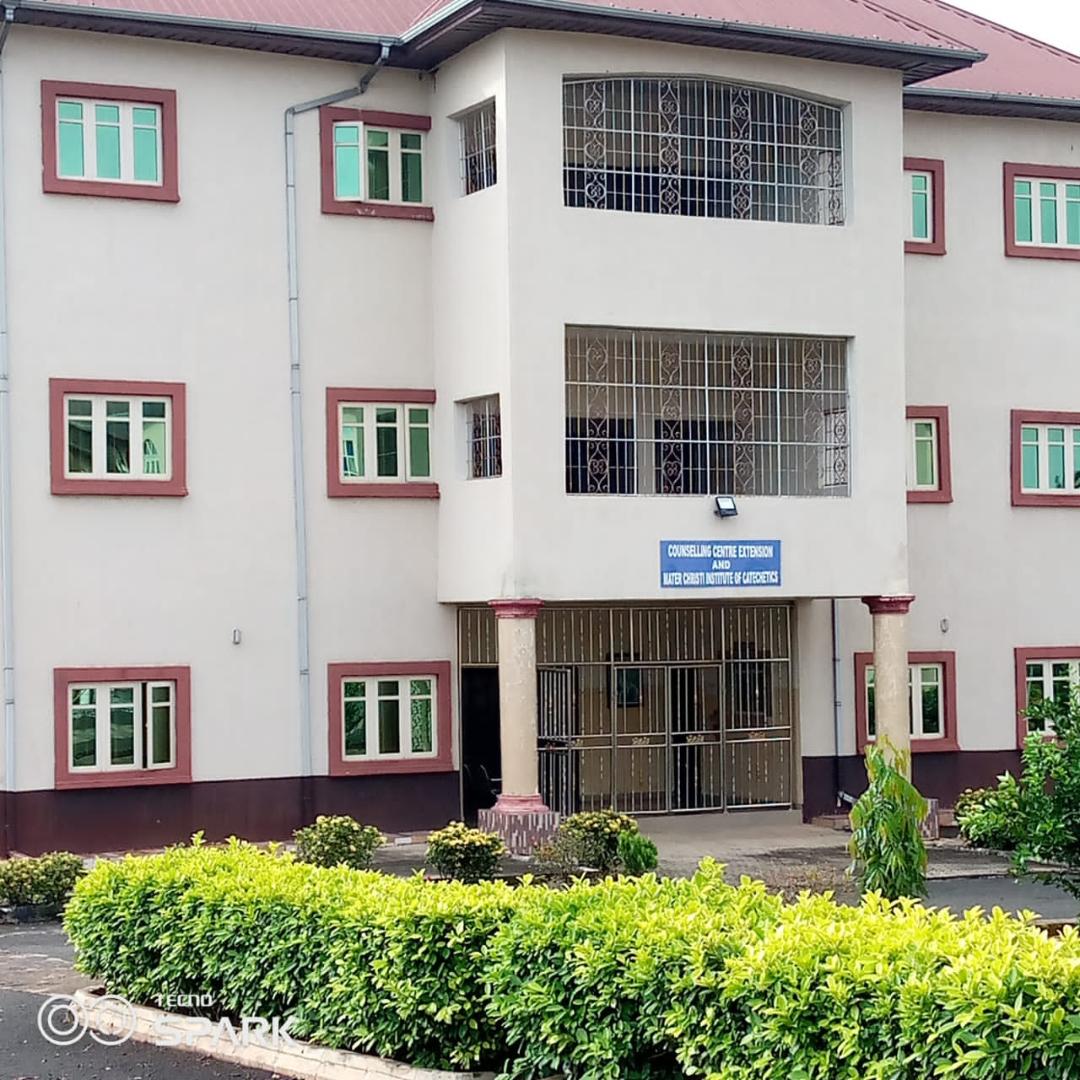** |
| --- |
| **The Front View of MACCARCA** |
| **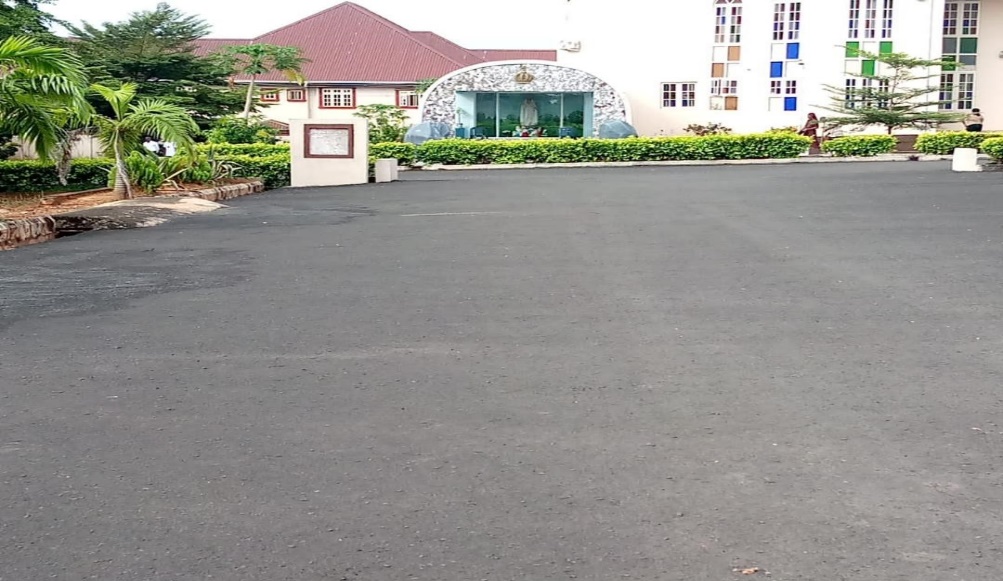** |
| **The Entrance of MACCARCA** |
| Figure 1: Images of MACCARCA therapy centre |

Supplement: Supplementary file 1 [file Table_1.docx]
